# Emotion Regulation and Neurocognitive Profiles in Adolescents With Selective Mutism

**DOI:** 10.1002/cpp.70261

**Published:** 2026-03-20

**Authors:** Celal Yesilkaya, Serkan Turan

**Affiliations:** ^1^ Department of Child and Adolescent Psychiatry Konya Ereğli State Hospital Konya Turkey; ^2^ Department of Child and Adolescent Psychiatry Uludağ University School of Medicine Bursa Turkey

**Keywords:** adolescence, behavioural problems, emotion regulation, neurocognition, selective mutism

## Abstract

While selective mutism (SM) is often conceptualized as a childhood anxiety disorder, longitudinal evidence suggests persistent difficulties into adolescence, yet the underlying cognitive and emotional mechanisms remain poorly understood. We aimed to address this gap by examining whether specific neurocognitive impairments and emotion regulation difficulties characterize adolescents with SM. Eighty‐nine adolescents (42 SM, 47 HC), aged 11–17 years, were assessed using the Strengths and Difficulties Questionnaire (SDQ), the Difficulties in Emotion Regulation Scale (DERS) and a neuropsychological battery evaluating attention, inhibition, processing speed, memory and social cognition. Adolescents with SM demonstrated greater emotional awareness difficulties (*p* = 0.010), with no differences in total DERS scores. SDQ scores indicated higher inattention/hyperactivity (*p* < 0.001), alongside significantly higher prosocial behaviour (*p* < 0.001). Neurocognitive findings revealed intact verbal learning but impaired immediate and delayed recall (*p* < 0.001), better delayed visual memory (*p* = 0.001). Children with SM made fewer total errors on Wisconsin Card Sorting Test (*p* = 0.021) and showed higher foil accuracy on CPT (*p* = 0.007), but demonstrated significantly poorer Stroop colour‐word (*p* = 0.001) and interference scores (*p* = 0.001). Adolescents with SM showed a distinctive cognitive–emotional profile, suggesting that internal emotional processing and external social sensitivity are dissociated during adolescence. Longitudinal and neurobiological research is warranted to elucidate mechanisms and refine treatment strategies.

## Summary

- Selective mutism (SM) in adolescence involves specific difficulties in emotional awareness and selective neurocognitive impairments rather than global deficits, requiring comprehensive assessment beyond speech behaviour.
- Adolescents with SM demonstrate preserved prosocial motivation and social cognition, suggesting interventions should leverage these strengths rather than focusing solely on deficits.
- Executive function profiles are heterogeneous, with preserved cognitive flexibility but impaired inhibitory control under pressure, indicating the need for targeted cognitive training in anxiety‐provoking contexts.
- Emotion regulation interventions should specifically target emotional awareness and recognition rather than broad‐based training, as total emotion dysregulation is not elevated in SM.

## 1 Introduction

Selective mutism (SM) is an anxiety disorder usually observed in childhood, characterized by the child's consistent failure to speak in certain settings where speaking is expected, despite speaking in other settings (Koskela et al. 2023). Previous research has reported the prevalence of SM to be between 0.18% and 1.9%, with an onset typically occurring between the ages of 3 and 6 (Viana et al. 2009).

Although SM is commonly described as an early‐onset condition, long‐term follow‐up studies have shown that while overt muteness often decreases with age, many individuals continue to experience significant social communication difficulties, heightened social anxiety and functional impairment during adolescence and beyond (Remschmidt et al. 2001; H.‐C. Steinhausen 2006). In adolescence, these difficulties may become particularly pronounced due to increasing social demands, peer interactions and academic expectations, contributing to persistent challenges in social participation, academic performance and emotional well‐being (Hans‐Christoph Steinhausen and Juzi 1996; H.‐C. Steinhausen 2006). Moreover, adolescents with a history of SM have been reported to exhibit elevated rates of internalizing psychopathology, including anxiety and depressive symptoms, as well as ongoing communication‐related avoidance, which may adversely affect peer relationships and psychosocial development (Remschmidt et al. 2001; H.‐C. Steinhausen 2006), highlighting that SM should not be regarded solely as a transient childhood condition, but rather as a disorder with potentially enduring developmental implications extending into adolescence.

Emotion regulation (ER) difficulties may be both a maintaining factor and a potential intervention target in SM. Adolescents with SM often exhibit challenges in identifying, understanding and modulating their emotions, which can contribute to avoidance behaviours in social and academic settings. Previous studies focusing on the emotional aspects of SM suggest that the condition is not merely a ‘not speaking problem’ but also involves significant emotional and behavioural regulation difficulties. According to recent research, parent reports indicate that children with SM have poorer ER skills compared to healthy controls (HCs) (Tenenbaum 2019). Additionally, another study found that children with SM display greater vigilance and more intense emotional reactions than both HC and children with Social Anxiety Disorder (Scott 2012). While most existing research on ER in SM has focused on younger children, emerging evidence suggests that ER profiles in adolescents with SM may differ in important ways. As ER becomes increasingly deliberate and cognitively mediated during adolescence, difficulties in SM appear to be less global and more strategy‐specific. A previous comparison study including both children and adolescents suggested that individuals with SM did not show deficits in overall adaptive or maladaptive ER, but rather exhibited reduced use of problem‐oriented behaviour and cognitive problem‐solving, alongside increased reliance on abandonment strategies (Melfsen et al. 2022). However, few studies have systematically evaluated self‐reported ER in SM populations, which is important, given the marked behavioural inhibition and limited verbal expression characteristic of SM; externally observable indicators may underestimate internal regulatory capacities. Self‐reported ER allows for the assessment of subjective emotional experiences and strategy use that may not be evident behaviourally, especially during adolescence when internal awareness and reflective regulation processes are more developed.

Similar to other anxiety disorders (Eysenck et al. 2007), individuals with SM exhibit neurocognitive impairments that may contribute to symptom maintenance and functional difficulties. Impairments in cognitive flexibility and working memory, as well as alterations in inhibitory control, have been specifically linked to the persistence of anxiety symptoms (Faber 2019), which may be particularly relevant for understanding the chronic nature of SM in adolescents. Previous research indicated that different domains of neurocognition, such as verbal memory (Kristensen and Oerbeck 2006; Manassis et al. 2007), processing speed (White 2019) and problem solving (Melfsen et al. 2022) were impaired in SM; conversely, attention was not significantly impaired (Oerbeck and Kristensen 2008). However, findings regarding visual memory were inconsistent (Kristensen and Oerbeck 2006; Manassis et al. 2007). Understanding these neurocognitive functions is particularly important in SM because they directly impact the core deficit of the disorder—the ability to speak in social situations. For instance, working memory and cognitive flexibility are essential for managing the cognitive demands of social interactions, while inhibitory control difficulties may contribute to the inability to override the avoidance response in speaking situations. Furthermore, in adolescence, when social demands and academic pressures intensify, these neurocognitive functions become increasingly critical for adaptive functioning. However, how these specific neurocognitive impairments relate to the severity of SM symptoms and functional outcomes in adolescents remains poorly understood, highlighting the need for comprehensive neurocognitive assessment in this population.

Despite increasing recognition of SM in clinical practice, research examining the cognitive and emotional profiles of affected adolescents remains limited. The present study aims to address these gaps by integrating parent‐reported behavioural assessments, adolescent self‐reported ER and objective neurocognitive measures in adolescents with SM and matched HCs. Specifically, we investigate (1) whether adolescents with SM demonstrate greater difficulties in ER compared to healthy peers, (2) whether neurocognitive performance differs between groups across attention, inhibition, psychomotor speed and learning/memory domains and (3) the associations between neurocognitive functioning, ER difficulties and behavioural problems. By integrating multiple sources of information, this study seeks to provide a comprehensive understanding of the cognitive and emotional mechanisms underlying SM, potentially informing targeted interventions and clinical assessment practices.

## 2 Method

### 2.1 Participants

The study sample included 89 adolescents between the ages of 10–18, 42 cases diagnosed with SM and 47 HC. The SM group had a mean age of 13.8 ± 2.1 years with 30 females (71.4%), while the control group had a mean age of 13.7 ± 2.1 years with 34 females (72.3%). Groups were comparable for age and gender distribution (see Table 1).

**TABLE 1:** Sociodemographic and illness characteristics of the study groups.

Variables	SM, *n* = 42	HC, *n* = 47	*t*/*x* ^2^	*p*
Age, y, M ± SD	13.8 ± 2.1	13.7 ± 2.1	0.2	0.811
Gender, female, *n* (%)	30 (71.4)	34 (72.3)	0.0	0.924
Maternal education			5.2	0.076
Elementary	19 (45.2)	11 (23.4)		
High school	7 (16.7)	14 (29.8)		
College	16 (38.1)	22 (46.8)		
Paternal education			0.1	0.932
Elementary	12 (28.6)	12 (25.5)		
High school	13 (31.0)	16 (34.0)		
College	17 (40.5)	19 (40.4)		
Medication				
Antipsychotics	8 (19.0)	—	—	—
Antidepressants	31 (73.8)	—	—	—
Comorbidity				
MDD	19 (45.2)	—	—	—
SpAD	9 (21.4)	—	—	—
SAD	11 (26.2)	—	—	—
GAD	12 (28.6)	—	—	—

Abbreviations: GAD, generalized anxiety disorder; HC, healthy controls; M, mean; MDD, major depressive disorder; SAD, social anxiety disorder; SD, standard deviation; SM, selective mutism; SpAD, separation anxiety disorder; *t*, independent samples *t*‐test; χ ^2^, chi‐square.

Participants with SM were recruited from a child and adolescent psychiatry outpatient clinic in Türkiye. All cases had an established diagnosis of SM documented in their clinical records and were either under ongoing follow‐up or newly referred to the clinic with a confirmed SM diagnosis at the time of recruitment. All participants underwent comprehensive clinical interviews using the ‘Kiddie Schedule for Affective Disorders and Schizophrenia for School‐Age Children—Present and Lifetime Version (K‐SADS‐PL, Turkish Version)’ (Ünal et al. 2019), conducted separately with children and parents by experienced child and adolescent psychiatrists, to confirm SM diagnosis according to The Diagnostic and Statistical Manual of Mental Disorders, fifth edition, text revision (DSM‐5‐TR) criteria and to identify or exclude current and past comorbid psychiatric conditions. The control group consisted of adolescents without psychiatric diagnoses or medication use from a similar epidemiological environment in Turkey, recruited from local schools, who volunteered through advertisements. Individuals with a history of head trauma, substance use, medical and neurological disorders that can have negative effects on cognitive functions, a history of psychotic disorders, bipolar disorder, intellectual disability and autism spectrum disorder that hindered maintaining neurocognitive assessments were excluded. The study protocol was reviewed and approved by the Local Ethics Committee of Uludağ University (2024/892‐16). Written informed consent was obtained from all participants and their families.

### 2.2 Measures and Outcome Variables

Sociodemographic data forms, completed jointly by adolescents and their families, were utilized to gather general information about the participants. The presence or absence of current and past psychiatric diagnoses was assessed using K‐SADS‐PL (Ünal et al. 2019), a semistructured interview administered to both adolescents and their families. Final diagnoses were established based on consensus among all authors. Participants' emotional and behavioural problems were assessed using the Strengths and Difficulties Questionnaire (SDQ)–Turkish version (Güvenir et al. 2008), completed by their parents. Emotion dysregulation was assessed using the self‐report Turkish version of the Difficulties in Emotion Regulation Scale (DERS) (Rugancı and Gençöz 2010).

Given the nature of SM and its potential influence on neurocognitive performance, specific procedures were implemented to ensure reliable assessment. Before the formal assessment, a 5‐ to 10‐min rapport‐building warm‐up phase was conducted to facilitate engagement. During this phase, adolescents were allowed to interact verbally or nonverbally at their own pace. If spontaneous speech did not occur, gradual verbal prompting and, when necessary, parent‐assisted fade‐in techniques were applied. Subtests requiring verbal responses were administered only after minimal verbal engagement was established.

To assess verbal learning and memory, processing speed, inhibition, attention, visual learning and memory and social cognition, all participants completed a comprehensive neuropsychological test battery administered by a trained researcher. Verbal learning and memory were assessed using the Rey Auditory Verbal Learning Test (RAVLT), with outcome variables including total learning across trials 1–5, immediate recall (trial 6) and delayed recall (trial 7), where higher scores indicate better performance. Processing speed was evaluated using the Digit–Symbol Subtest of the Wechsler Intelligence Scale for Children–Revised (WISC‐R) and the Stroop Word subtest, with higher scores reflecting faster processing speed. Inhibitory control was assessed using the Stroop Word–Colour subtest and the interference score was calculated, with higher interference scores indicating poorer inhibition. Attention and response inhibition were examined using a continuous performance test (CPT). Primary outcome variables included target accuracy, reflecting sustained attention and foil accuracy, reflecting correct inhibition of responses to target stimuli, with higher scores indicating better performance. Visual learning and memory were assessed using the immediate and delayed recall scores of the Visual Reproduction subtest of the Wechsler Memory Scale, with higher scores indicating better visual memory performance. Social cognition was assessed using the Reading the Mind in the Eyes Test (RMET), and total correct responses were used as the outcome measure. Stroop test and CPT were administered through computer. The duration of the neurocognitive assessment ranged from 45 to 60 min based on individual participant performance. All analyses were conducted using raw scores.

### 2.3 Statistical Analysis

A priori power analysis was conducted using G*power v3.1.9.7 (Faul et al. 2009) to test differences between two independent group means using a two‐tailed test. Based on a study in the previous literature suggesting a moderate effect size (*d* = 0.55) (Ciray and Turan 2024), power analysis indicated a minimum number of 42 participants for each group (*ß* = 0.80, α = 0.05, allocation ratio = 1).

The normality of data distribution was assessed using kurtosis and skewness values. Continuous variables with normal distribution are reported as mean ± standard deviation. Group differences in neurocognitive test and scale scores were analyzed using analysis of covariance (ANCOVA), controlling for maternal education and the presence of major depressive disorder (MDD) comorbidity because of the high comorbidity in the SM group.

To derive a single global cognitive score, a factor analysis was conducted on the neurocognitive test variables. Principal component analysis was used with the number of components fixed at two, followed by Oblimin rotation. Component scores were saved as regression scores, and a composite Global Cognition variable was created by averaging the two component scores. The two‐component solution accounted for 51.9% of the total variance. A multiple linear regression analysis was performed to identify factors associated with global cognition in adolescents with SM. The dependent variable was global cognition score, while independent variables included gender, presence of comorbidity, SDQ‐Peer relationships subscore, presence of family psychiatric diagnosis and total score on the DERS.

Statistical significance was set at *p* < 0.05. Statistical analyses were performed using IBM SPSS (Statistical Package for Social Sciences; SPSS Inc., Chicago, IL, USA) 26.0.

## 3 Results

A total of 42 adolescents with SM and 47 HCs were included in the study. The SM and control groups were comparable in terms of age (13.8 ± 2.1 vs. 13.7 ± 2.1 years, *p* = 0.811), gender (*p* = 0.924) and paternal education (*p* = 0.932). Maternal education levels showed a trend towards difference between groups (*p* = 0.076). Among the cases with SM, psychiatric comorbidities were common, with major depressive disorder being the most frequent (45.2%), followed by generalized anxiety disorder (28.6%), social anxiety disorder (26.2%) and separation anxiety disorder (21.4%). By definition, the control group had no psychiatric diagnoses. Regarding medication use, 73.8% of SM cases were receiving antidepressants and 19.0% were on antipsychotics, while controls were medication‐free. Sociodemographical and clinical characteristics of the participants are summarized in Table 1.

A significant multivariate effect of group was detected (Pillai's Trace = 0.578, *p* < 0.001). Therefore, follow‐up ANCOVAs were conducted to examine group differences on each neurocognitive variable. In the verbal domain, while RAVLT‐ learning scores showed a trend‐level difference (*p* = 0.070), the individuals with SM demonstrated significantly poorer immediate recall (*p* = 0.002) and delayed recall performance (*p* < 0.001) compared to HC. Cases with SM showed comparable immediate visual reproduction (*p* = 0.202) but significantly impaired delayed visual recall (*p* = 0.001). For social cognition, no significant difference was found between groups on the RMET, though a trend‐level effect was observed (*p* = 0.063). Regarding processing speed, digit‐symbol test performance was comparable between groups (*p* = 0.357), but the cases with SM was significantly slower in Stroop word reading (*p* = 0.004). Both stroop colour‐word performance (*p* = 0.001) and Stroop interference scores (*p* = 0.001) were significantly impaired in adolescents with SM compared to HC. On the WCST, while the number of correct responses showed a trend‐level difference (*p* = 0.066), the SM group made significantly more total errors (*p* = 0.021) compared to controls. No significant differences were found in perseverative responses (*p* = 0.716) or perseverative errors (*p* = 0.126). Individuals with SM demonstrated significantly lower foil accuracy on the CPT (*p* = 0.007), while target accuracy showed a trend‐level difference (*p* = 0.073). All significant findings remained robust after applying false discovery rate (FDR) correction using the Benjamini–Hochberg method (*q* < 0.05). Neurocognitive performances of the case group and HC are shown in Table 2.

**TABLE 2:** Neurocognitive test comparisons of the groups.

Neurocognitive test scores	SM, *n*	HC, *n*	*F*	*p*	Interpretation	*q* (FDR)
**Verbal domain**						
RAVLT						
Learning (1–5)	42	42	3.4	0.070	Trend‐level	0.102
Immediate recall	42	42	9.7	**0.002**	SM < HC	**0.006**
Late recall (7)	42	42	20.8	**< .001**	SM < HC	**0.016**
**Visual domain**						
Visual reproduction						
Immediate recall	42	42	1.7	0.202	Not significant	0.229
Late recall	42	42	12.0	**0.001**	SM < HC	**0.008**
**Social cognition**						
RMET	42	42	3.6	0.063	Trend‐level	0.112
**Processing speed**						
Digit‐symbol	42	42	1.1	0.357	Not significant	0.379
Stroop word	42	42	8.8	**0.004**	SM < HC	**0.011**
**Problem solving**						
WCST						
Correct responses	42	42	3.5	0.066	Trend‐level	0.106
Total error	42	42	5.5	**0.021**	SM>HC	**0.042**
Per. responses	42	42	0.1	0.716	Not significant	0.716
Per. errors	42	42	2.4	0.126	Not significant	0.154
**Inhibition**						
Stroop‐colour word*	42	42	15.8	**0.001**	SM < HC	**0.005**
Stroop interference*	42	42	10.8	**0.001**	SM < HC	**0.004**
**Attention**						
CPT‐accuracy						
Target accuracy, %	42	42	3.3	0.073	Trend‐level	0.097
Foil accuracy, %	42	42	7.6	**0.007**	SM > HC	**0.016**

*Note:* FDR *q* values calculated using Benjamini–Hochberg correction (*q* < 0.05).

Abbreviations: ANCOVA, adjusted for maternal education and MDD comorbidity; CPT, continuous performance task; HC, healthy controls; Per., perseverative; RAVLT, Rey Auditory Verbal Learning Test; RMET, reading mind in the eyes test; SM, selective mutism; t, independent samples *t*‐test; WCST, Wisconsin Card Sorting Test.

* Logarithmic transformation.

The results of the parent‐reported SDQ, adjusted for maternal education and MDD comorbidity, revealed significant group differences in specific domains that remained significant after FDR correction. Adolescents with SM had significantly higher inattention/hyperactivity scores (*p* < 0.001) and significantly higher prosocial behaviour scores (*p* < 0.001) compared to HC. No significant differences were found in conduct problems (*p* = 0.844), emotional problems (*p* = 0.487), peer problems (*p* = 0.135) or the total difficulty score (*p* = 0.115). Regarding DERS scores, cases with SM showed significantly higher difficulties in emotional awareness (*p* = 0.010) compared to controls. The clarity subscale showed a trend‐level difference (*p* = 0.077). No significant differences were found in the goals (*p* = 0.113), strategy (*p* = 0.776), impulsivity (*p* = 0.603) or nonacceptance subscales (*p* = 0.520) or in the total DERS score (*p* = 0.544). The comparisons of the DERS and parent‐reported SDQ scores between the groups are displayed in Table 3.

**TABLE 3:** Comparison of SDQ and DERS scores between groups.

	SM, *n*	HC, *n*	*F*	*p*	Interpretation	*q* (FDR)
SDQ						
Inattention/hyperactivity	42	42	15.0	**< 0.001**	SM > HC	**0.013**
Conduct problems	42	42	0.0	0.844	Not significant	0.844
Emotional problems	42	42	0.5	0.487	Not significant	0.791
Peer problems	42	42	2.3	0.135	Not significant	0.251
Prosocial behaviour	42	42	132.0	**< 0.001**	SM > HC	**0.007**
Total difficulty score	42	42	2.5	0.115	Not significant	0.249
DERS						
Goal	42	42	2.6	0.113	Not significant	0.294
Strategy	42	42	0.1	0.776	Not significant	0.841
Impulsivity	42	42	0.3	0.603	Not significant	0.713
Awareness	42	42	7.0	**0.010**	SM > HC	**0.043**
Clarity	42	42	3.2	0.077	Trend‐level	0.250
Nonacceptance	42	42	0.4	0.520	Not significant	0.751
Total	42	42	0.4	0.544	Not significant	0.707

Abbreviations: ANCOVA, adjusted for maternal education and MDD comorbidity; DERS, Difficulties in Emotion Regulation Scale; SDQ, Strengths and Difficulties Questionnaire; SM, selective mutism; *t*, independent samples *t*‐test.

The multiple linear regression model which performed to identify factors associated with global cognition in adolescents with SM was statistically significant (*F*(5, 35) = 3.114, *p* = 0.020), accounting for 30.8% of the variance in global cognition scores. The analysis revealed that gender emerged as a significant predictor (*β* = 0.41, *p* = 0.007), with higher global cognition scores observed among males. Additionally, peer relationships demonstrated a significant positive association with global cognition (*β* = 0.31, *p* = 0.042). Other variables in the model did not reach statistical significance. The multiple regression analysis can be found in Table 4.

**TABLE 4:** Multiple regression analysis investigating predictor variables for global cognition in adolescents with selective mutism (*N* = 42).

Variables	*β* (95% CI)	*p*
Gender	0.41 (0.15 to 0.67)	**0.007**
Presence of comorbidity	0.10 (−0.30 to 0.49)	0.507
SDQ‐peer relationships	0.31 (0.01 to 0.60)	**0.042**
Psychiatric diagnosis in Family	−0.24 (−0.70 to 0.23)	0.120
DERS total score	0.12 (−0.37 to 0.61)	0.407

*Note*: R^2^ = 0.31; adjusted *R* ^2^ = 0.21.

Abbreviations: β, standardized beta coefficient; CI, confidence interval; DERS, Difficulties in Emotion Regulation Scale; SDQ, Strengths and Difficulties Questionnaire.

## 4 Discussion

This study aimed to investigate ER, behavioural functioning and neurocognitive performance in adolescents with SM compared to HC. Our findings provide evidence that SM is a multifaceted condition characterized by specific ER challenges and selective neurocognitive alterations rather than a uniform cognitive deficit profile. These results contribute to an emerging body of literature that conceptualizes SM as an anxiety spectrum disorder with unique developmental and neurobehavioural underpinnings (Muris and Ollendick 2021).

Difficulties in ER have long been hypothesized as a maintaining factor in SM (Melfsen et al. 2022; Scott 2012; Tenenbaum 2019). In line with this framework, our findings revealed significant group differences in emotional awareness, with adolescents with SM demonstrating greater difficulties in recognizing and attending to internal emotional states, consistent with prior reports of heightened vigilance and emotional reactivity (Melfsen et al. 2022; Milic et al. 2023; Tenenbaum 2019). Such deficits at early stages of emotional processing may perpetuate avoidance behaviours in social and performance‐related contexts by constraining access to adaptive regulation strategies. In contrast, emotional clarity was only elevated at a trend level, suggesting that once emotional states are identified, individuals with SM may be relatively less impaired in differentiating or interpreting their emotions. Together, these findings support a nuanced ER profile in SM, characterized by primary difficulties in emotional awareness rather than pervasive deficits across later‐stage interpretive processes.

Contrary to expectations, total DERS scores did not differ significantly between groups, underscoring that ER difficulties in SM are selective rather than pervasive. This finding parallels the results of Melfsen et al. (2022), who reported that SM was associated with lower use of problem‐oriented strategies without generalized ER impairment (Melfsen et al. 2022). Together, these findings suggest that targeted interventions focusing on emotional awareness and goal‐directed action may be more effective than broad ER training for adolescents with SM.

Parent‐reported SDQ scores indicated elevated inattention/hyperactivity in adolescents with SM, in line with literature documenting attentional challenges as secondary consequences of mutism (Melfsen et al. 2022; Zakszeski and DuPaul 2017). This elevation may reflect the cognitive demands of managing chronic anxiety in social situations, which could deplete attentional resources and manifest as inattentive or hyperactive behaviours in daily settings. A striking and somewhat counterintuitive finding was significantly higher prosocial behaviour in the SM group. While previous studies have emphasized social withdrawal and avoidance, our data suggest that SM adolescents may possess strong social motivation and affiliative tendencies, even when verbal communication is inhibited. This aligns with recent evidence that many children with SM actively seek social inclusion and demonstrate positive nonverbal communication strategies (Vogel et al. 2024), and the conceptualization of SM as rooted in anxiety rather than a lack of social interest (Cohan et al. 2008). Thus, prosocial behaviour may represent a compensatory channel through which these adolescents express their social motivation.

Our neurocognitive results reveal a complex pattern characterized by both vulnerabilities and strengths. In the verbal domain, adolescents with SM demonstrated poorer immediate and delayed recall, suggesting intact encoding with compromised consolidation or retrieval. This discrepancy may reflect heightened cognitive effort during initial exposure due to anxiety, followed by interference effects that impair recall—consistent with attentional control theory, which posits that anxiety disrupts working memory and retrieval processes (Eysenck et al. 2007; Faber 2019). Notably, these findings partially diverge from earlier reports of broad verbal memory deficits (Kristensen and Oerbeck 2006; Manassis et al. 2007), likely due to age differences and comorbidity profiles in our sample.

Executive functioning findings were heterogeneous. WCST performance indicated better problem‐solving accuracy with fewer total errors in the SM group, suggesting preserved cognitive flexibility in structured tasks. However, this contrasted with impaired inhibitory control evident in Stroop tasks, where SM children required significantly longer completion times for both colour‐word naming and interference conditions. This pattern suggests selective executive vulnerabilities, particularly in inhibition under time pressure and interference conditions, consistent with prior research linking anxiety disorders to compromised attentional control (Eysenck et al. 2007; Faber 2019). Interestingly, CPT results revealed enhanced foil accuracy in SM, indicating superior ability to correctly reject nontarget stimuli. This heightened discriminative attention may reflect a compensatory mechanism or hypervigilant processing style, which could contribute to their cautious approach in social contexts but becomes maladaptive when rapid, automatic responses are required.

Adolescents with SM demonstrated a trend towards higher performance on the RMET compared to HCs, which should be interpreted cautiously. This finding suggests that theory of mind may be relatively preserved or even heightened in SM. Such a pattern aligns with conceptualizations of SM as a disorder characterized not by deficits in social cognition per se, but by heightened social sensitivity and hypervigilance to interpersonal cues (Muris and Ollendick 2021). In contrast to autism spectrum disorder, where impairments in affective theory of mind are well documented, individuals with SM may retain an intact—or even heightened—particularly in evaluative social contexts. Together with the other neurocognitive test findings, these results form a coherent pattern. On the CPT, the case group showed a trend towards higher target accuracy and significantly better foil accuracy, which indicates preserved sustained attention and better response inhibition. Together, these findings suggest an overcontrolled cognitive‐affective profile in SM with high vigilance and cautious responding. Thus, SM may be better conceptualized not as a disorder of social‐cognitive deficit, but as one marked by excessive control and inhibition that constrains adaptive emotional expression.

When considered alongside the observed difficulties in emotional awareness, this pattern points to a dissociation between internal and external socio‐emotional processing, which is, adolescents with SM may struggle to recognize and regulate their own internal emotional states while simultaneously being attuned to others' emotions. Such an imbalance aligns with developmental models of social anxiety suggesting that heightened awareness of others' mental states, in the absence of adequate internal emotional insight or regulation, may exacerbate self‐consciousness, fear of evaluation and avoidance behaviours (Colonnesi et al. 2017). Rather than reflecting a protective factor, relatively preserved or heightened theory of mind may therefore contribute to the maintenance of SM by amplifying perceived social threat.

Our multiple regression analysis revealed that gender and peer relationship quality emerged as significant predictors of global cognition, providing important insights about factors that may influence cognitive outcomes in this understudied clinical population. Male gender was associated with higher global cognition scores compared to their female counterparts. This finding is particularly noteworthy, given that SM is more commonly diagnosed in females (Muris and Ollendick 2021), and the majority of our sample (71.4%) consisted of girls. This gender difference in cognitive performance may reflect several underlying mechanisms: First, it is possible that females with SM experience more severe symptomatology or co‐occurring internalizing problems that interfere with cognitive performance during assessment. Previous research has suggested that females with anxiety disorders often exhibit higher rates of comorbid mood disorders (McLean et al. 2011) and may experience greater functional impairment (Lewinsohn et al. 1995). Alternatively, the gender difference could reflect differences in compensatory strategies or neurobiological factors affecting cognitive processing in the context of severe social anxiety. Alternatively, the gender difference could reflect differences in compensatory strategies or neurobiological factors affecting cognitive processing in the context of SM. The positive association between higher SDQ‐Peer Problems scores and better global cognition was unexpected. This counterintuitive finding may reflect the parent‐reported nature of the measure, as parental perceptions of social functioning can sometimes diverge from adolescents' actual cognitive performance, potentially contributing to this pattern.

Our findings underscore that SM is not merely a disorder of speech but a condition involving integrated emotional, cognitive and behavioural processes. Critically, it is important to distinguish speech from language: While speech enables auditory expression, expressive language encompasses vocabulary, grammar, pragmatic understanding and the formulation of meaning—all of which represent distinct cognitive demands implicated in SM. Clinical interventions should address ER deficits (particularly emotional awareness) and inhibitory control while leveraging cognitive strengths and prosocial motivation. Exposure‐based therapies might be optimized by incorporating ER‐focused components, mindfulness strategies and inhibitory control training. Furthermore, emerging evidence supports innovative approaches—such as teletherapy integrating vocal physiology and pragmatic language training—which have demonstrated promising outcomes in adolescents with SM (Klein and Ruiz 2025).

From a research perspective, these findings invite deeper exploration of neural mechanisms underlying SM. Recent meta‐analytic work on ER (Monachesi et al. 2023) highlights the role of shared prefrontal networks in implementing inhibitory and regulatory strategies, suggesting that dysfunction in these circuits may underlie SM's behavioural phenotype. Future studies should integrate neuroimaging with longitudinal designs to clarify whether inhibitory control deficits represent a risk factor, a maintaining mechanism or both. Additionally, future research should incorporate standardized, norm‐referenced measures of expressive language, as verbal output in the present study was assessed only at the word level through verbal memory tasks. Given that more advanced language skills have been associated with better ER in children (Fields‐Olivieri et al. 2024), expressive language represents an important variable for building a more complete neurocognitive profile of SM.

## 5 Limitations

This study's limitations include its cross‐sectional design, which precludes causal inference, and the potential influence of psychotropic medication use in the SM group on cognitive performance. Although depressive symptoms were statistically controlled, future studies with larger samples are needed to better clarify how comorbid conditions may independently or jointly influence cognitive functioning and ER in SM. The sample size, though adequate for detecting medium effect sizes, limits generalizability, particularly regarding gender differences or subtype analysis. Another limitation concerns the use of the WISC‐R Digit Symbol subtest, as the WISC‐V has not yet been standardized for the Turkish population. Although the WISC‐R norms are outdated, this subtest represented the most accessible and clinically applicable measure available at the time of data collection. Furthermore, speech latency and hesitation, which are core characteristics of SM, may have influenced Stroop test performance. Because the Stroop task requires verbal responses, the observed slowness in word reading among adolescents with SM may partially reflect speech‐related hesitation rather than purely cognitive processing differences. This represents a potential confound that should be addressed in future studies using computerized or manual response versions of the Stroop task. The multiple regression analysis included five predictor variables with a sample of 41 participants, which falls below the commonly recommended ratio of 15–20 observations per predictor. This limits the statistical reliability and generalizability of the regression findings, and replication with larger samples is warranted. Finally, while self‐report ER measures offer valuable subjective insight, they may not fully capture implicit regulatory processes; future studies should incorporate multimodal assessments, including physiological and neuroimaging indices.

## 6 Conclusion

This study shows that SM in adolescence is a multidimensional condition involving selective ER difficulties and specific neurocognitive characteristics rather than being solely a speech disorder. These findings highlight the importance of interventions that combine behavioural strategies with ER and cognitive flexibility training. Future research should use longitudinal and neurobiological approaches to clarify mechanisms and identify predictors of treatment response.

## Ethics Statement

The Local Ethics Committees of Uludag University reviewed and approved the study protocol (2024/892‐16). All procedures performed in studies involving human participants were in accordance with the ethical standards of the institutional and/or national research committee and with the 1964 Helsinki declaration and its later amendments or comparable ethical standards.

## Consent

Written informed consent was obtained from all participants and their families.

## Data Availability

All data and materials used in this study are available on request from the corresponding author.
